# Supplementary material for: Cerebrospinal Fluid Concentrations of Meropenem and Vancomycin in Ventriculitis Patients Obtained by TDM-Guided Continuous Infusion
Source: Antibiotics (Basel). 2021 Nov 20;10(11):1421. doi: 10.3390/antibiotics10111421 (PMC8614961; doi:10.3390/antibiotics10111421)
Supplement: Supplementary file 1 [file antibiotics-10-01421-s001.zip › antibiotics-1456461-supplementary.pdf]

Supplemental Materials

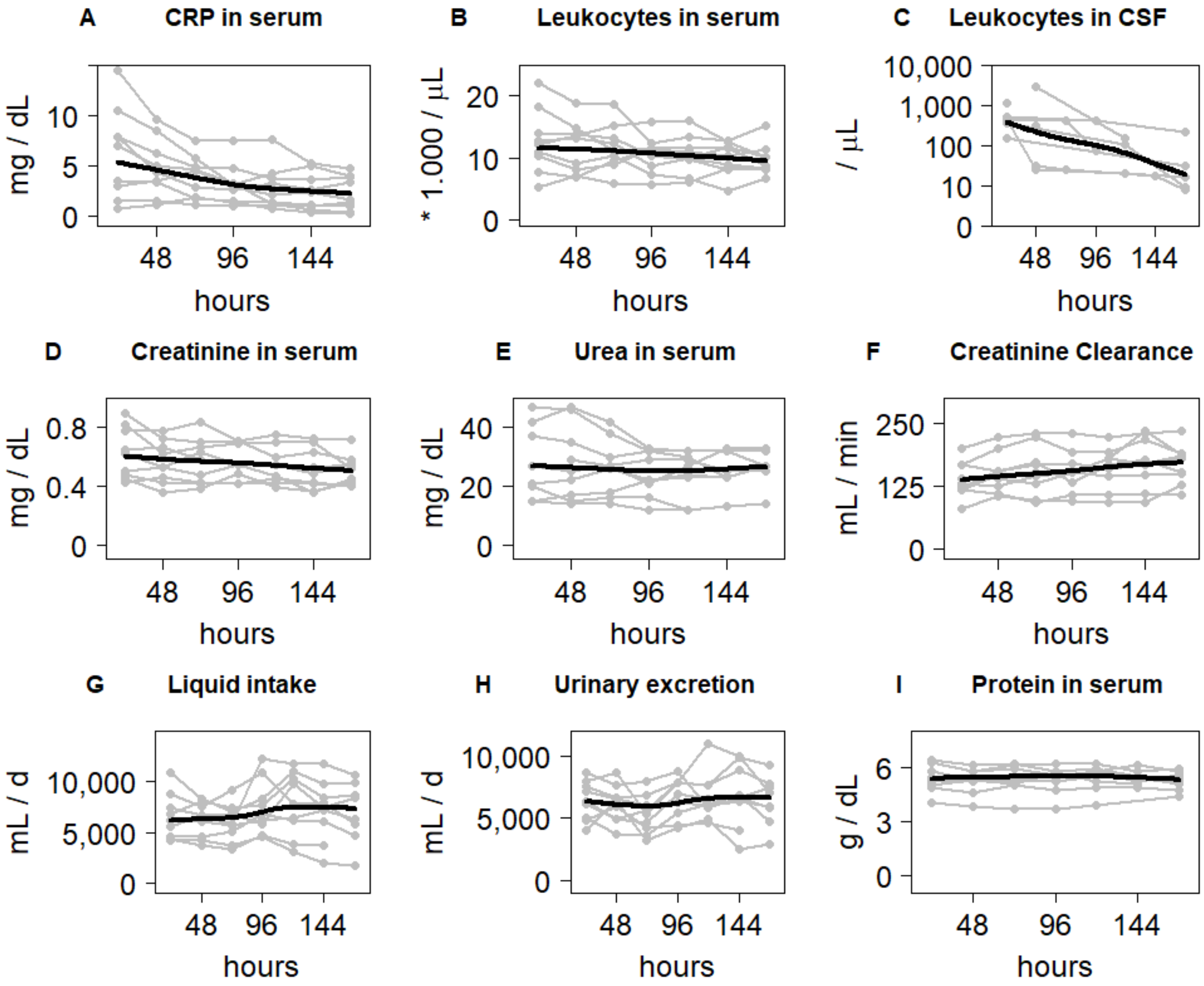

Figure S1. Parameters of inflammation and possible confounding variables of drug concentrations. **A-C** C-reactive protein, together with leukocyte count in both, serum and CSF, decreased during antibiotic therapy. **D-F** All patients presented with normal renal retention parameters throughout the treatment period. Noteworthy, no renal impairment could be detected despite high-dose vancomycin therapy. **G-H** Fluid intake and urinary excretion balanced at 6 L/d and remained unchanged during therapy. **I** No difference in whole protein concentrations in serum was observed over the course of treatment.

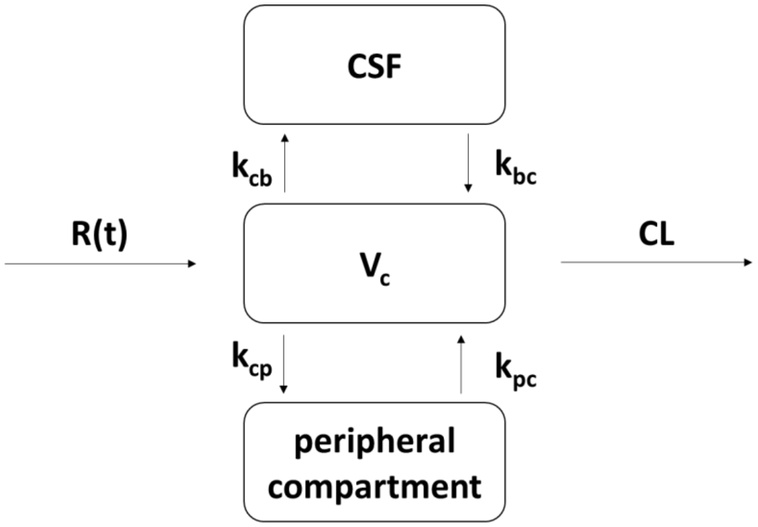

Figure S2. Pharmacokinetic model. V_c in L represents the volume of the central compartment. R(t) infusion rate, CL clearance, k_{cp}, k_{pc}, k_{bc} and k_{cb} in h^{-1} represent first-order transfer constants connecting the various compartments. k_{bc} describes the transition from CSF to the central compartment, while accordingly k_{cb} stands for the transition from the central compartment to the CSF, k_{pc} for the transition from the peripheral compartment to the central compartment and k_{cp} for the transition from the central compartment to the peripheral compartment.
